# Who is more likely to receive up-to-date lung cancer screening? Identifying key barriers using principal component analysis and SHAP modeling: a weighted cross-sectional analysis of the 2024 BRFSS

**DOI:** 10.3389/fpubh.2026.1803742

**Published:** 2026-06-18

**Authors:** Shanshan Li, Jinxian He, Fang Li, Chao Jin, Hui Tian

**Affiliations:** Thoracic Surgery, The Affiliated Lihuili Hospital of Ningbo University, Ningbo, Zhejiang, China

**Keywords:** BRFSS, cluster analysis, lung cancer screening, SHAP, social determinants of health

## Abstract

**Background:**

Up-to-date lung cancer screening (UTD-LCS) uptake remains low. This cross-sectional study examines how social determinants and physical or mental well-being are associated with UTD-LCS using multiple analytical methods with appropriate survey weighting.

**Methods:**

We analyzed 6,234 eligible adults aged 50–79 from the 2024 Behavioral Risk Factor Surveillance System (BRFSS), applying complex survey weights throughout all analyses. Principal Component Analysis (PCA) was used to combine 14 social determinants and health indicators into latent dimensions. Partitioning Around Medoids (PAM) clustering stratified the population into distinct subgroups based on the optimal number of clusters determined by silhouette coefficients, and SHapley Additive exPlanations (SHAP) were employed to visualize the specific predictors of screening uptake within each cluster. A sensitivity analysis was conducted excluding adults aged 78–79 to assess the potential implications of the Medicare coverage gap on screening behaviors.

**Results:**

The weighted UTD-LCS uptake was 20.0% (SE = 1.1%). PCA identified four components explaining 56.0% of the variance: Socioeconomic Resources, Physical Health, Psychosocial Health, and Employment & Neighborhood. In the fully adjusted weighted logistic regression, only Socioeconomic Resources was independently associated with screening (OR = 0.822, 95% CI: 0.713–0.948, *p* = 0.007); Physical Health showed a borderline positive association (OR = 1.126, *p* = 0.053), while Psychosocial Health and Employment & Neighborhood were not significant. PAM clustering identified two groups—a General Population cluster (*n* = 5,435; screening rate 20.2%) and an Employment/Neighborhood Vulnerable cluster (*n* = 799; screening rate 18.7%)—with no significant difference between groups (*p* = 0.579). SHAP analysis revealed that Physical Health was the top predictor in both clusters, but in the Vulnerable cluster, the predictive importance of Physical Health and Socioeconomic Resources was substantially amplified. Sensitivity analysis excluding adults aged 78–79 confirmed Socioeconomic Resources significance and rendered Physical Health significant (*p* = 0.032), suggesting that the Medicare coverage gap attenuates the physical health–screening association.

**Conclusion:**

Socioeconomic deprivation is the primary barrier to UTD-LCS. While poor physical health prompts screening, the Medicare coverage gap may potentially hinder this pathway.

## 1 Introduction

Lung cancer remains the leading cause of cancer-related mortality worldwide and in the United States (1). Early detection through low-dose computed tomography (LDCT) has been shown to reduce lung cancer mortality by approximately 20% among high-risk populations (2–4). In response to this evidence, the U.S. Preventive Services Task Force (USPSTF) updated its guidelines in 2021 to expand eligibility by lowering the screening age from 55 to 50 and reducing the smoking history requirement from 30 to 20 pack-years (5, 6). Despite these expanded criteria and the established survival benefits, screening uptake remains alarmingly low, with national estimates indicating that only 16 to 18% of eligible individuals receive screening (7, 8).

Understanding the barriers to up-to-date lung cancer screening (UTD-LCS) is critical for improving participation rates. While demographic factors such as age, insurance status, and race are well-documented predictors (9–13), recent research has increasingly recognized that individual screening behaviors are deeply embedded in a broader context of social determinants and physical and mental well-being. Emerging evidence indicates that adverse social determinants—such as financial instability, lack of transportation, and social isolation—alongside poor physical and mental health status, constitute significant barriers to preventive care (14–16). However, most prior studies have tended to examine these indicators in isolation or relied on conventional regression analyses that assume uniform effects across populations. Moreover, many studies using BRFSS data have not appropriately accounted for the complex survey design, potentially producing biased estimates and inflated Type I error rates. This “one-size-fits-all” analytical approach often fails to capture the complex, multidimensional interactions between socioeconomic deprivation and physical or psychosocial health, potentially masking distinct risk profiles within the eligible population.

Therefore, this study focuses on discerning “who is more or less likely to undergo lung cancer screening” by integrating associated factors into two comprehensive dimensions: Social Determinants (comprising 10 indicators) and Physical/Mental Health (comprising 4 indicators). Utilizing data from the 2024 Behavioral Risk Factor Surveillance System (BRFSS) with appropriate complex survey weighting, we utilized Principal Component Analysis, survey-weighted logistic regression, and clustering methods to identify factors associated with screening participation, aiming to inform targeted public health interventions.

## 2 Methods

### 2.1 Data source and study participants

This study utilized data from the 2024 BRFSS, a premier cross-sectional health-related telephone survey conducted annually by the Centers for Disease Control and Prevention (CDC) in collaboration with state health departments across the United States (17). Data were collected from January 1 through December 31, 2024, and accessed for this analysis in March 2026. The BRFSS collects data from non-institutionalized U.S. adults aged 18 years and older using random-digit-dialing techniques on both landlines and cellular telephones. The survey utilizes a complex multi-stage sampling design to ensure the data is representative of the U.S. population. As this study involved the analysis of a publicly available, de-identified dataset, it was deemed exempt from review by the Institutional Review Board. This study was reported following the Strengthening the Reporting of Observational Studies in Epidemiology (STROBE) guidelines for cross-sectional studies.

To account for the complex survey design, all analyses incorporated the BRFSS survey design variables: stratification variable (_STSTR), primary sampling unit (_PSU), and the final person-level weight for the lung cancer screening module (_LLCPWT), following CDC guidelines for the analysis of optional BRFSS modules.

**Figure 1:**
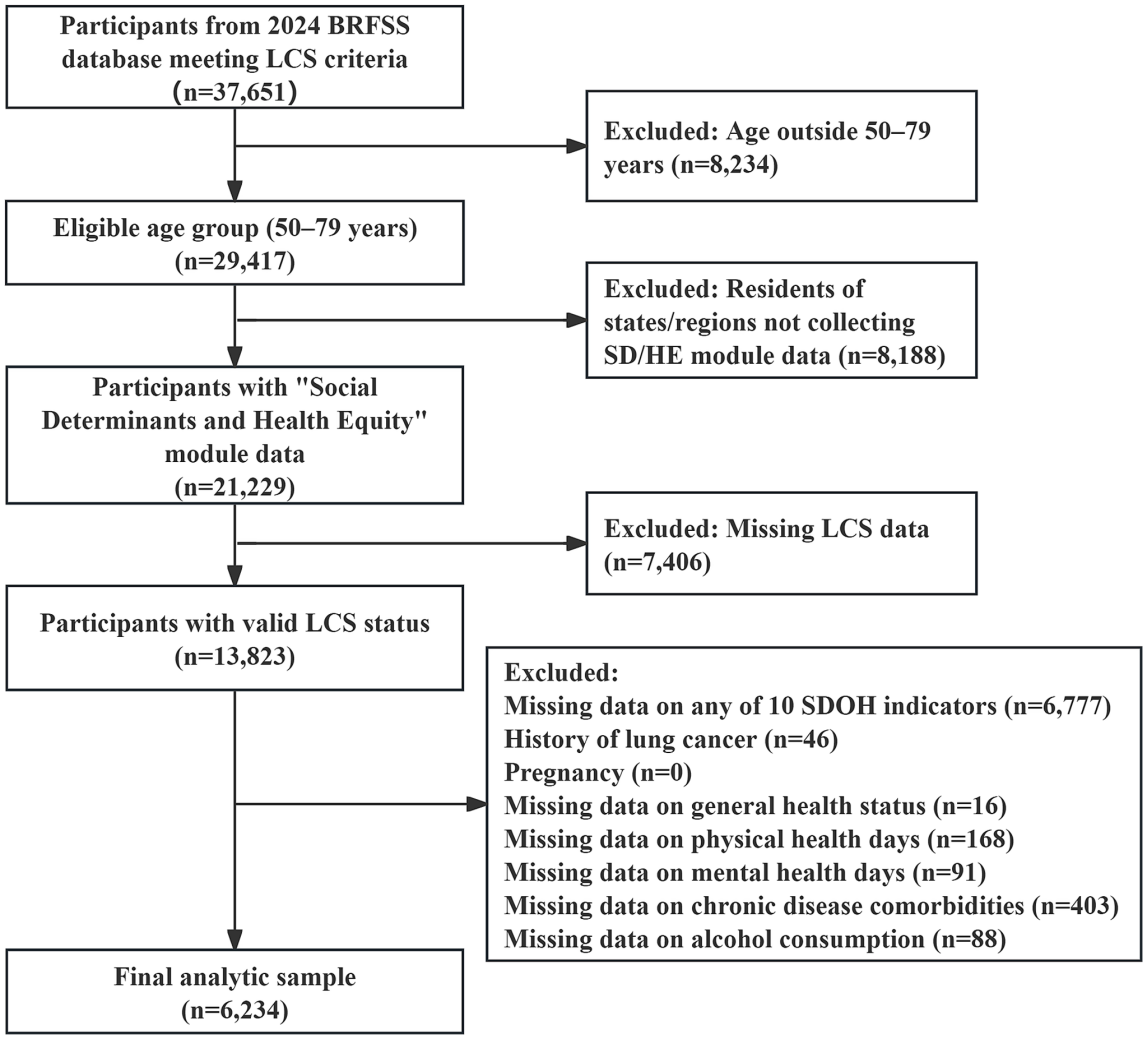
Flowchart of participants selection.

The participant selection process is illustrated in Figure 1. Initially, we identified 37,651 participants who met the high-risk smoking criteria for LCS (persons who currently smoke or formerly smoked and who quit within the past 15 years, with at least a 20 pack-year smoking history) (18, 19). We restricted the analysis to individuals aged 50 to 79 years (*n* = 29,417). Although the USPSTF guidelines recommend screening up to age 80, participants aged 80 and older were excluded because the BRFSS aggregates age into a single category (“80+”) for this group (18) preventing precise age-based adjustments. Since the “Social Determinants and Health Equity” (SD/HE) module is an optional module chosen by specific jurisdictions, we excluded 8,188 participants from 11 states that did not collect this data (Arkansas, Colorado, Hawaii, Illinois, Louisiana, Maryland, Michigan, Nebraska, New York, North Dakota, and South Dakota), leaving 21,229 participants. We further applied sequential exclusion criteria based on data completeness: (1) missing data on LCS status (*n* = 7,406); (2) missing data on any of the 10 key social determinants of health indicators (*n* = 6,777); (3) a self-reported history of lung cancer (*n* = 46); and (4) pregnancy at the time of survey (*n* = 0). We then excluded individuals with missing values for general health status (n = 16), physical health (*n* = 168), mental health (*n* = 91), or any of the 10 chronic disease comorbidities (*n* = 403). Finally, 88 participants with missing alcohol consumption data were excluded following reclassification of the alcohol variable (see Section 2.3). The final analytic sample consisted of 6,234 eligible participants.

### 2.2 Outcome and exposure variables

The primary outcome of this study was up-to-date lung cancer screening (UTD-LCS), defined as undergoing a computed tomography (CT) scan specifically for screening purposes within the past 12 months. This binary outcome was derived from two sequential questions in the BRFSS survey. First, participants who reported ever having a CT scan were asked, “Were any of the CT or CAT scans of your chest area done mainly to check or screen for lung cancer?” Second, those who answered affirmatively were asked to specify the timing of their most recent scan. Participants were classified as having UTD-LCS only if they confirmed the scan was for screening purposes and reported that it occurred “within the past year (anytime less than 12 months ago).” All other eligible participants—including those who had never been screened, those screened for diagnostic purposes, or those screened more than 12 months prior—were classified as non-UTD-LCS. It is important to note an inherent limitation of the BRFSS survey instrument at this stage: because it asks generally about “CT or CAT scans,” it cannot definitively distinguish whether respondents received a true guideline-concordant low-dose computed tomography (LDCT) or a standard diagnostic chest CT. Consequently, this variable serves as a self-reported proxy for screening behavior.

The study examined 14 exposure variables categorized into two primary domains: social determinants and health status. We utilized 10 indicators directly from the BRFSS “Social Determinants” module to capture socioeconomic and environmental context, including life satisfaction, frequency of social and emotional support, frequency of loneliness, employment instability (lost job or reduced hours), receipt of food stamps (SNAP), food insecurity (difficulty affording to buy the things needed), difficulty paying bills, difficulty paying utility costs, lack of reliable transportation, and perceived neighborhood safety. Additionally, four physical and mental health indicators were included based on recent literature (15): general health status, the number of days with poor physical health in the past 30 days, the number of days with poor mental health in the past 30 days, and the aggregate burden of chronic diseases, calculated as the count of 11 self-reported chronic conditions (including heart disease, coronary heart disease, stroke, asthma, skin cancer, other types of cancer, chronic lung disease, depression, kidney disease, arthritis, and diabetes).

### 2.3 Covariates

We collected sociodemographic characteristics and health-related behaviors as potential covariates. Demographic variables included age, sex (Male, Female), race/ethnicity (categorized as Non-Hispanic White, Non-Hispanic Black, Non-Hispanic Other, Non-Hispanic Multiracial, and Hispanic), educational attainment (Less than high school, Graduated high school, Attended college or technical school, Graduated from college or technical school), marital status (Married, Others), annual household income (<$50,000, ≥$50,000), and residence type (Urban counties, Rural counties). Health behaviors and clinical characteristics included smoking status (person who smokes daily, person who smokes some days, person who formerly smoked), age of smoking initiation, body mass index (BMI < 25 kg/m^2^, BMI ≥ 25 kg/m^2^), health insurance coverage (Yes, No), and participation in leisure-time physical activity (Yes, No). Alcohol consumption was assessed using a three-category classification: non-drinker (no alcohol consumption in the past 30 days), moderate drinker (consumed alcohol but did not meet criteria for binge or heavy drinking), and binge/heavy drinker (met criteria for binge drinking and/or heavy drinking based on CDC definitions). This reclassification addressed the prior conflation of abstainers with moderate drinkers and resulted in the exclusion of 88 participants with missing alcohol data.

### 2.4 Statistical analysis

All analyses accounted for the BRFSS complex survey design. A survey design object was specified incorporating stratification (_STSTR), primary sampling units (_PSU), and person-level weights (_LLCPWT). Data are presented as weighted median [interquartile range, IQR] for continuous variables and weighted *n* (%) for categorical variables. Baseline characteristics of the study population were compared between those who underwent UTD-LCS and those who did not, using Rao-Scott chi-square tests for categorical variables and design-based Mann–Whitney tests. All statistical tests were two-sided, and a *p*-value of less than 0.05 was considered statistically significant. To assess potential selection bias introduced by participant exclusions, we compared the baseline characteristics of included and excluded populations using Standardized Mean Differences (SMDs) and design-adjusted tests. An absolute SMD (|SMD|) ≥ 0.1 was used to identify meaningful systematic imbalances.

To address the potential multicollinearity and information redundancy among the 14 indicators covering social determinants and physical/mental health, we employed Principal Component Analysis (PCA) on the weighted correlation matrix. The suitability of the data for factorization was assessed using the Kaiser-Meyer-Olkin (KMO) test and Bartlett’s Test of Sphericity. We retained components with eigenvalues greater than 1 and applied Varimax rotation, retaining factor loadings with an absolute value greater than 0.5 as the primary contributors. Following this dimension reduction, we calculated individual standardized factor scores for each of the identified components for every participant. These continuous factor scores were then used as the primary independent variables in survey-weighted multivariable logistic regression models (‘svyglm’) to investigate the associations between these latent component scores and screening behavior. In Model 1, each component score was analyzed individually, adjusting for 12 covariates (age, sex, race/ethnicity, education, household income, BMI category, health insurance, marital status, urban/rural residence, smoking status, leisure-time exercise, and alcohol consumption). In Model 2, all four component scores were entered simultaneously along with all 12 covariates to determine independent effects. Let $p_{i}$ denote the probability that individual i undergoes up-to-date lung cancer screening (UTD-LCS = 1). The log-odds (logit) of screening was modeled as follows:

Model 1:


$$\ln(\frac{p_{i}}{1-p_{i}})=\alpha^{\left(j\right)}+\beta_{\mathrm{PC}}^{\left(j\right)}PC_{\mathrm{ji}}+\sum_{k=1}^{12}\gamma_{k}^{\left(j\right)}C_{\mathrm{ki}}$$

Model 2:


$$\ln(\frac{p_{i}}{1-p_{i}})=\alpha +\beta_{1}PC_{1i}+\beta_{2}PC_{2i}+\beta_{3}PC_{3i}+\beta_{4}PC_{4i}+\sum_{k=1}^{12}\gamma_{k}C_{\mathrm{ki}}$$

α is the intercept; $PC_{\mathrm{ji}}$ represents the standardized continuous principal component score for individual i on the j -th component; $C_{\mathrm{ki}}$ represents the k -th covariate for individual i ; β and γ represent the regression coefficients for the principal component scores and covariates, respectively. Instead of including survey weights as independent predictors, the parameters ( α,β,γ ) in both models were estimated by maximizing a survey-weighted pseudo-log-likelihood function:


$$\ln L(\theta )=\sum_{i=1}^{n}w_{i}[y_{i}\ln(p_{i})+(1-y_{i})\ln(1-p_{i})]$$

where $y_{i}\in \{0,1\}$ is the observed binary screening outcome, and $w_{i}$ is the final person-level survey weight (_LLCPWT) corresponding to individual i . This approach, computed using the svyglm function with a quasibinomial link, ensures unbiased population-level coefficient estimates and correctly adjusted standard errors that incorporate stratification (_STSTR) and primary sampling units (_PSU). To assess the robustness of our multivariable logistic regression results, we conducted a sensitivity analysis using multiple imputation by chained equations (MICE) to evaluate the impact of missing data on effect estimates.

Furthermore, we classified the study population using Partitioning Around Medoids (PAM) clustering based on the four derived principal component scores. The optimal number of clusters was determined by evaluating silhouette coefficients across k = 2 to 6, selecting the k with the highest average silhouette width. We compared the weighted UTD-LCS uptake across the identified clusters using the Rao-Scott chi-square test. Finally, to reveal the heterogeneous drivers of screening behavior among different socially vulnerable groups, we trained separate XGBoost models within each cluster and utilized SHapley Additive exPlanations (SHAP) to quantify the specific contribution of each component’s factor score to the screening decision within each identified cluster. It is important to note that SHAP values quantify the conditional contribution of predictors to the model’s prediction; they are purely predictive in nature and do not establish causal or structural relationships. To appropriately account for the BRFSS sampling design within the machine learning framework, person-level survey weights were mean-normalized and explicitly incorporated into the XGBoost data matrix to weight the logistic loss function during training. Because our primary objective was model interpretability rather than pure predictive deployment, hyperparameters were constrained to prevent overfitting and ensure the stability of SHAP values. Specifically, we utilized a shallow tree architecture with a maximum depth of 3, a learning rate of 0.1, a subsampling rate of 0.8, and 100 boosting rounds. Model discrimination and calibration were evaluated using survey-weighted Area Under the Curve (AUC) and weighted accuracy. A sensitivity analysis was performed by excluding participants aged 78–79 years to assess the impact of the Medicare coverage gap, as the Centers for Medicare & Medicaid Services currently covers lung cancer screening only for individuals aged 50 to 77 years. All analyses were performed using R version 4.5.3 (20). Complex survey designs were incorporated using the survey package (21). Machine learning and model interpretability were implemented using the xgboost (22) and shapviz (23) packages.

## 3 Results

### 3.1 Characteristics of the study population

A total of 6,234 eligible participants were included in the final analysis, representing an estimated 3,209,182 weighted individuals in the U.S. population. Among them, 1,525 (weighted 20.0%, SE = 1.1%) reported undergoing UTD-LCS within the past year, while 4,709 (weighted 80.0%) did not (Table 1). This survey-weighted screening rate of 20.0% aligns closely with recent national estimates (7, 8). Notably, 2,194 participants (weighted 35.3%) reported ever having a CT scan for lung cancer screening purposes, while only 1,525 (weighted 20.0%) met the UTD-LCS definition. This gap suggests that approximately 15.3% of eligible adults have been screened at some point but are not currently adherent to annual screening recommendations, indicating a substantial retention challenge distinct from initial uptake barriers. The screened group had a significantly higher median number of cigarettes smoked per day. Regarding the specific exposure variables related to social determinants, participants who underwent UTD-LCS were significantly less likely to report difficulty paying bills (9.7% vs. 13.5%, *p* = 0.024) or difficulty paying utility costs (7.1% vs. 12.0%, *p* = 0.001).

**Table 1:** Baseline characteristics of the study population stratified by lung cancer screening status (weighted).

Variable	Total (*N* = 6,234)	Non-UTD-LCS (*n* = 4,709)	UTD-LCS (*n* = 1,525)	*p*
Age, year	64 [59–70]	64 [59–70]	64 [59–70]	0.482
Age started smoking, years	16 [14–18]	16 [14–18]	16 [14–18]	0.864
Cigarettes per day	20 [12–20]	20 [10–20]	20 [15–20]	0.0188
Sex, *n* (%)				0.255
Male	3,251 (51.9%)	2,461 (51.2%)	790 (54.5%)	
Female	2,983 (48.1%)	2,248 (48.8%)	735 (45.5%)	
Race, *n* (%)				0.738
White only, Non-Hispanic	5,287 (83.8%)	3,984 (83.6%)	1,303 (84.5%)	
Black only, Non-Hispanic	373 (6.5%)	279 (6.4%)	94 (6.9%)	
Other race only, Non-Hispanic	184 (3.5%)	140 (3.8%)	44 (2.3%)	
Multiracial, Non-Hispanic	111 (2.3%)	83 (2.3%)	28 (2.4%)	
Hispanic	202 (4%)	161 (4%)	41 (4%)	
Education level, *n* (%)				0.749
Less than high school	553 (11.2%)	408 (11.5%)	145 (10.2%)	
Graduated high school	2,217 (35.6%)	1,677 (35.6%)	540 (35.4%)	
Attended college or technical school	2,110 (33.3%)	1,607 (32.8%)	503 (35.2%)	
Graduated from college or technical school	1,346 (19.9%)	1,009 (20.1%)	337 (19.1%)	
BMI, n (%)				0.902
<25 kg/m^2^	1,910 (31.5%)	1,407 (31.4%)	503 (31.7%)	
≥25 kg/m^2^	4,140 (68.5%)	3,166 (68.6%)	974 (68.3%)	
Annual income, *n* (%)				0.961
<$50,000	3,210 (56.4%)	2,455 (56.4%)	755 (56.5%)	
≥$50,000	2,318 (43.6%)	1,728 (43.6%)	590 (43.5%)	
Health insurance, *n* (%)				0.082
No	163 (3.1%)	140 (3.4%)	23 (1.8%)	
Yes	5,924 (96.9%)	4,460 (96.6%)	1,464 (98.2%)	
Leisure time exercise, *n* (%)				0.205
No	2,429 (40.1%)	1,824 (40.8%)	605 (37.3%)	
Yes	3,793 (59.9%)	2,875 (59.2%)	918 (62.7%)	
Urban or Rural status, *n* (%)				0.578
Urban	5,127 (85.1%)	3,870 (84.9%)	1,257 (86.1%)	
Rural	1,038 (14.9%)	781 (15.1%)	257 (13.9%)	
Smoking status, *n* (%)				0.24
Person who smokes daily	2,979 (48.1%)	2,255 (48%)	724 (48.5%)	
Person who smokes some days	503 (8.1%)	367 (7.6%)	136 (9.9%)	
Person who formerly smoked	2,752 (43.8%)	2087 (44.4%)	665 (41.6%)	
Marital status, *n* (%)				0.194
Married	2,813 (47.3%)	2,103 (46.6%)	710 (49.9%)	
Others	3,402 (52.7%)	2,591 (53.4%)	811 (50.1%)	
Alcohol use, *n* (%)				0.8
Non-drinker	3,570 (56.6%)	2,687 (56.6%)	883 (56.6%)	
Moderate	623 (10%)	463 (9.8%)	160 (10.9%)	
Binge/Heavy drinking	2041 (33.4%)	1,559 (33.7%)	482 (32.5%)	
General health, *n* (%)				0.699
Excellent	285 (4.2%)	210 (4.2%)	75 (4.1%)	
Very good	1,219 (19.2%)	936 (19.1%)	283 (19.7%)	
Good	2,213 (34.2%)	1,672 (34.9%)	541 (31.5%)	
Fair	1,649 (27.6%)	1,245 (27.3%)	404 (28.6%)	
Poor	868 (14.8%)	646 (14.5%)	222 (16.2%)	
Physical health (days not good), *n* (%)				0.8
0 days	2,902 (43.7%)	2,223 (43.9%)	679 (42.7%)	
1–13 days	1,431 (24%)	1,090 (24.1%)	341 (23.6%)	
≥14 days	1,901 (32.3%)	1,396 (32%)	505 (33.7%)	
Chronic disease burden, count	2 [1–4]	2 [1–4]	2 [1–4]	0.26
Mental health (days not good), *n* (%)				0.84
0 days	3,645 (55.4%)	2,760 (55.3%)	885 (56.2%)	
1–13 days	1,363 (22.8%)	1,020 (22.7%)	343 (23.1%)	
≥14 days	1,226 (21.8%)	929 (22.1%)	297 (20.7%)	
Life satisfaction, *n* (%)				0.0518
Very satisfied	2,142 (33.6%)	1,608 (33.1%)	534 (35.8%)	
Satisfied	3,481 (55.2%)	2,621 (55.3%)	860 (54.9%)	
Dissatisfied	453 (7.8%)	352 (7.7%)	101 (7.8%)	
Very dissatisfied	158 (3.4%)	128 (3.9%)	30 (1.4%)	
Emotional support, *n* (%)				0.592
Never	357 (6.4%)	268 (6.6%)	89 (5.3%)	
Rarely	400 (6.4%)	315 (6.6%)	85 (5.4%)	
Sometimes	939 (14.5%)	729 (14.8%)	210 (13.5%)	
Usually	1,546 (25.6%)	1,151 (25%)	395 (27.7%)	
Always	2,992 (47.2%)	2,246 (46.9%)	746 (48.1%)	
Loneliness, *n* (%)				0.724
Never	2,358 (38.9%)	1,779 (38.8%)	579 (39.2%)	
Rarely	1,758 (28.6%)	1,323 (28.9%)	435 (27.6%)	
Sometimes	1,587 (24.5%)	1,201 (24.7%)	386 (23.9%)	
Usually	275 (4.1%)	207 (3.8%)	68 (5.3%)	
Always	256 (3.8%)	199 (3.8%)	57 (4%)	
Lost job or reduced hours, *n* (%)				0.944
No	5,758 (92.2%)	4,329 (92.3%)	1,429 (92.1%)	
Yes	476 (7.8%)	380 (7.7%)	96 (7.9%)	
Receiving food stamps, *n* (%)				0.0543
No	5,202 (83.2%)	3,914 (82.6%)	1,288 (86%)	
Yes	1,032 (16.8%)	795 (17.4%)	237 (14%)	
Food insecurity, *n* (%)				0.0699
Never	4,408 (69.9%)	3,289 (68.9%)	1,119 (74%)	
Rarely	682 (11.2%)	517 (11.5%)	165 (10.2%)	
Sometimes	623 (9.9%)	480 (9.9%)	143 (9.8%)	
Usually	211 (3.7%)	177 (4.1%)	34 (2.1%)	
Always	310 (5.3%)	246 (5.7%)	64 (3.9%)	
Bill payment difficulty, *n* (%)				0.0243
No	5,501 (87.3%)	4,133 (86.5%)	1,368 (90.3%)	
Yes	733 (12.7%)	576 (13.5%)	157 (9.7%)	
Utility payment difficulty, *n* (%)				0.00124
No	5,651 (89%)	4,240 (88%)	1,411 (92.9%)	
Yes	583 (11%)	469 (12%)	114 (7.1%)	
Lack of transportation, *n* (%)				0.251
No	5,611 (89.7%)	4,231 (89.3%)	1,380 (91.2%)	
Yes	623 (10.3%)	478 (10.7%)	145 (8.8%)	
Neighborhood safety a, *n* (%)				0.112
No	370 (5.8%)	287 (6.1%)	83 (4.4%)	
Yes	5,864 (94.2%)	4,422 (93.9%)	1,442 (95.6%)	

Data are presented as median [IQR] for continuous variables and n (%) for categorical variables. IQR, interquartile range; BMI, body mass index; UTD-LCS, up-to-date lung cancer screening.

A comparison of baseline characteristics between the included (*n* = 6,234) and excluded (*n* = 31,417) cohorts is presented in Supplementary Table 1. Systematic differences were observed; included participants were generally older (mean age: 63.5 vs. 57.7 years, SMD = 0.566), more likely to have health insurance (96.5% vs. 91.7%, SMD = 0.208), and more likely to be married (51.8% vs. 45.7%, SMD = 0.122). These differences were largely attributable to structural constraints, including state-level administration of the SDOH module and USPSTF age eligibility criteria.

### 3.2 Identification of latent components of social determinants and health status

PCA was conducted to identify latent structures among the 14 exposure indicators. The suitability of the data for dimensionality reduction was confirmed prior to the analysis. As shown in Table 2, the KMO measure of sampling adequacy was 0.846, indicating meritorious sampling adequacy. Bartlett’s Test of Sphericity was significant (chi-square = 19,638.2, df = 91, *p* < 0.001), rejecting the null hypothesis that the correlation matrix was an identity matrix and thus confirming that the variables were sufficiently correlated for PCA.

**Table 2:** KMO and Bartlett’s test (weighted correlation matrix).

Measure	Value
Kaiser-Meyer-Olkin Measure of Sampling Adequacy	0.846
Bartlett’s Test of Sphericity	
Approx. Chi-Square	19638.2
df	91
Sig.	<0.001

Principal component analysis was used as the extraction method, with components retained based on the Kaiser criterion of having eigenvalues greater than 1. This resulted in the extraction of four distinct components. The scree plot, presented in Table 3, visually confirmed a clear inflection point after the fourth component, supporting the four-component solution. These four components collectively explained 56.0% of the total variance in the dataset.

**Table 3:** Total variance explained by principal component analysis.

Component	Eigenvalue	% of Variance	Cumulative %
1	4.074	29.097	29.097
2	1.538	10.986	40.083
3	1.22	8.716	48.799
4	1.014	7.24	56.039
5	0.946	6.756	62.796
6	0.789	5.639	68.435
7	0.696	4.973	73.408
8	0.673	4.806	78.213
9	0.634	4.53	82.744
10	0.596	4.258	87.001
11	0.525	3.751	90.752
12	0.475	3.395	94.147
13	0.44	3.14	97.287
14	0.38	2.713	100

Extraction method: principal component analysis.

The four components were interpreted and labeled based on the variables with factor loadings greater than 0.50 after Varimax rotation, as detailed in Table 4. Component 1, labeled Socioeconomic Resources, was characterized by variables related to economic instability and resource deprivation, including bill payment difficulty (0.77), food insecurity (0.72), utility payment difficulty (0.72), receipt of food stamps (0.64), and lack of transportation (0.55). Component 2, labeled Physical Health, was dominated by the number of days with poor physical health (0.80), general health status (0.79), and chronic disease burden (0.72). Component 3, labeled Psychosocial Health, comprised emotional support (−0.79), loneliness (0.72), life satisfaction (0.65), and the number of days with poor mental health (0.57). Component 4, labeled Employment & Neighborhood, was defined by employment instability (0.73), perceived neighborhood safety (−0.71), and neighborhood safety (−0.71).

**Table 4:** Social determinants and health status and respective factor loadings for the four identified components.

Variables	Component 1	Component 2	Component 3	Component 4
	Socioeconomic resources	Physical health	Psychosocial health	Employment & Neighborhood
Life satisfaction			0.646	
Emotional support			−0.786	
Loneliness			0.718	
Lost job or reduced hours				0.732
Receiving food stamps	0.637			
Bill payment difficulty	0.772			
Utility payment difficulty	0.719			
Lack of transportation	0.549			
Neighborhood safety				−0.714
Food insecurity	0.722			
General health		0.791		
Physical health		0.798		
Mental health			0.57	
Chronic disease burden		0.718		

Extraction method: principal component analysis. Rotation method: varimax with Kaiser Normalization. Only factor loadings with |value| ≥ 0.50 are shown.

### 3.3 Association between latent components and UTD-LCS

Table 5 presents the results of survey-weighted logistic regression analyses examining the associations between the identified principal components and UTD-LCS uptake. The findings from both modeling approaches converged to highlight the primacy of socioeconomic factors.

**Table 5:** Association between four different principal component scores and LCS uptake.

Variable	Model 1: separate analysis^a^		Model 2: simultaneous analysis^b^	
	OR (95% CI)	*p*-value	OR (95% CI)	*p*-value
Principal component scores				
Socioeconomic resources	0.821 (0.710, 0.949)	0.008	0.822 (0.713, 0.948)	0.007
Physical health	1.147 (1.016, 1.294)	0.026	1.126 (0.998, 1.271)	0.053
Psychosocial health	0.961 (0.842, 1.096)	0.552	0.946 (0.831, 1.077)	0.403
Employment & Neighborhood	0.959 (0.849, 1.083)	0.504	0.948 (0.839, 1.073)	0.399

OR, odds ratio; CI, confidence interval; LCS, lung cancer screening.

a Model 1 represents four separate survey-weighted logistic regression models. Each component was entered individually, adjusted for age, education, BMI, insurance status, and smoking status.

b Model 2 represents a single survey-weighted logistic regression model including all four components, and all covariates simultaneously.

In Model 1, which assessed each latent component individually after adjusting for 12 covariates, Socioeconomic Resources (C1) was significantly associated with reduced odds of screening (OR = 0.821, 95% CI: 0.710–0.949, *p* = 0.008), and Physical Health (C2) showed a significant positive association (OR = 1.147, 95% CI: 1.016–1.294, *p* = 0.026), suggesting that persons with greater physical morbidity were more likely to undergo screening—likely reflecting their more frequent contact with the healthcare system. Psychosocial Health (C3: OR = 0.961, *p* = 0.552) and Employment & Neighborhood (C4: OR = 0.959, *p* = 0.504) were not statistically significant in the individual models.

When all four latent components were evaluated simultaneously in Model 2, only Socioeconomic Resources remained independently significant (OR = 0.822, 95% CI: 0.713–0.948, *p* = 0.007). Physical Health retained a borderline positive association (OR = 1.126, 95% CI: 0.998–1.271, *p* = 0.053), narrowly missing conventional significance after accounting for the complex survey design. Psychosocial Health (OR = 0.946, *p* = 0.403) and Employment & Neighborhood (OR = 0.948, *p* = 0.399) were not significant in the multivariable model.

### 3.4 Clustering and heterogeneous patterns of screening drivers

Building on the regression findings, which established socioeconomic deprivation as the primary barrier, we next examined whether the population could be meaningfully segmented into subgroups with distinct screening patterns. PAM clustering based on the four principal component scores was used, with the optimal number of clusters determined by silhouette coefficients. The analysis yielded k = 2 as the optimal solution (silhouette width = 0.437), substantially exceeding k = 3 (0.290) and all higher values, indicating that the population is best characterized by two rather than three or more subgroups.

**Figure 2:**
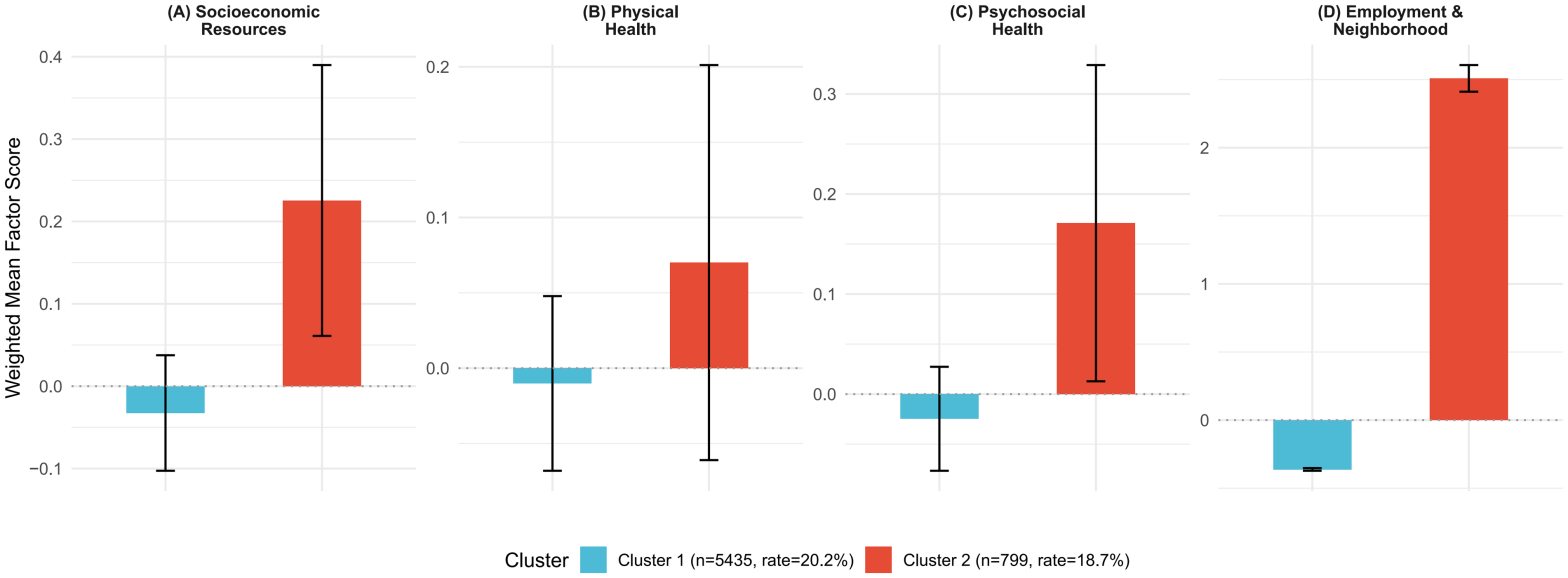
Distribution of weighted mean factor scores for the four components across the two population clusters identified by PAM analysis. **(A)** Socioeconomic resources; **(B)** Physical health; **(C)** Psychosocial health; **(D)** Employment & Neighborhood. Note: Bar charts display weighted mean factor scores with standard error bars. Clusters derived using PAM with k = 2 (silhouette = 0.437; k = 3: 0.290).

Figure 2 illustrates the mean factor scores for each component across the two clusters. Cluster 1 (*n* = 5,435; 87.2%), labeled the “General Population” group, exhibited scores close to the overall mean across all four dimensions, with a mildly low score on the Employment & Neighborhood component (mean = −0.36). Cluster 2 (*n* = 799; 12.8%), labeled the “Employment/Neighborhood Vulnerable” group, was characterized by an extremely elevated score on C4 (mean = 2.51), representing persons experiencing employment instability in neighborhoods perceived as unsafe, while scores on the remaining three dimensions were close to the population mean.

The weighted UTD-LCS rates were 20.2% (SE = 1.2%) for Cluster 1 and 18.7% (SE = 2.4%) for Cluster 2, with no statistically significant difference between groups (Rao-Scott chi-square = 0.880, *p* = 0.579). This null finding is coherent with the regression results, which demonstrated that Employment & Neighborhood (C4)—the primary dimension distinguishing the two clusters—was not independently associated with screening uptake. Prior to SHAP extraction, the performance of the XGBoost models was evaluated. The model for the General Population cluster achieved a weighted AUC of 0.794 and a weighted accuracy of 0.818. Similarly, the model for the Employment/Neighborhood Vulnerable cluster yielded a weighted AUC of 0.947 and a weighted accuracy of 0.917, indicating satisfactory discrimination for interpretative purposes.

**Figure 3:**
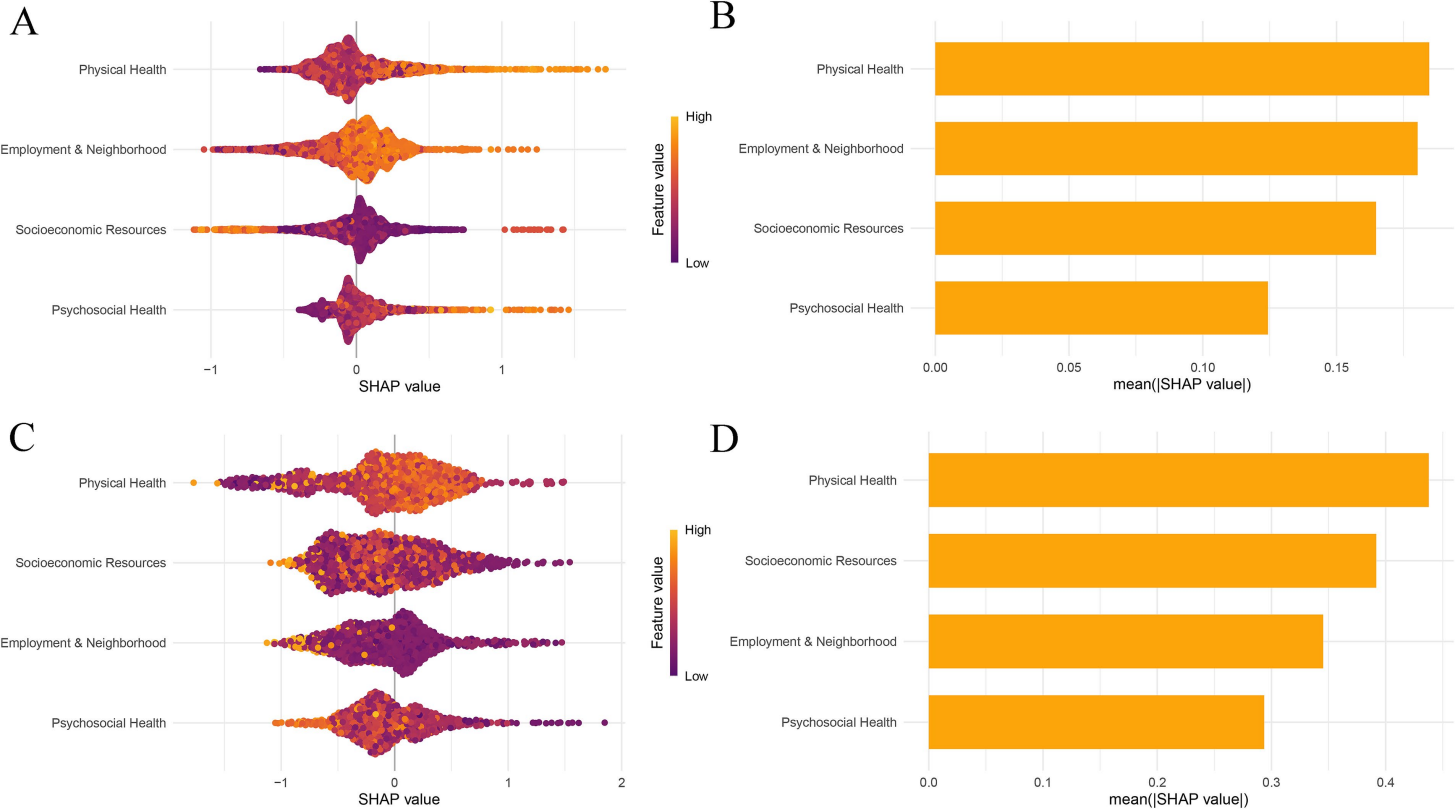
SHAP analysis illustrating the drivers of UTD-LCS behavior across the two identified population clusters. **(A,B)** Cluster 1—General Population; **(C,D)** Cluster 2—Employment/Neighborhood Vulnerable. Left panels **(A,C)** are SHAP beeswarm plots. Each dot represents a single participant. The position on the *x*-axis indicates the SHAP value: a positive SHAP value (right of the vertical zero line) implies an increased predictive probability of undergoing screening, whereas a negative value implies a decreased probability. The color of the dot represents the actual feature value (i.e., the standardized principal component score), with orange indicating a high score and purple indicating a low score. For example, in Panel **A**, high Physical Health scores (orange dots) are positioned to the right, indicating they positively predict screening. Right panels **(B,D)** are Mean |SHAP| bar charts, which display the average absolute SHAP value for each feature, representing its overall magnitude of predictive importance within that specific cluster. LCS, lung cancer screening; SHAP, SHapley Additive exPlanations.

Despite similar screening rates, SHAP analysis revealed informative differences in the predictive patterns operating within each cluster (Figure 3). For the General Population cluster (Cluster 1; Figures 3A,B), the four components showed relatively balanced predictive importance: Physical Health (mean |SHAP| = 0.185) ranked first, followed closely by Employment & Neighborhood (0.180), Socioeconomic Resources (0.165), and Psychosocial Health (0.124). In the Employment/Neighborhood Vulnerable cluster (Cluster 2; Figures 3C,D), all SHAP magnitudes were substantially amplified: Physical Health (0.438) and Socioeconomic Resources (0.392) dominated, followed by Employment & Neighborhood (0.345) and Psychosocial Health (0.294). This 2 to 3-fold amplification indicates that among persons experiencing employment instability and neighborhood insecurity, both physical health status and economic capacity demonstrate a markedly stronger predictive contribution on screening decisions—even though these intensified predictive weights do not translate into significantly different aggregate screening rates between groups.

### 3.5 Sensitivity analyses

**Figure 4:**
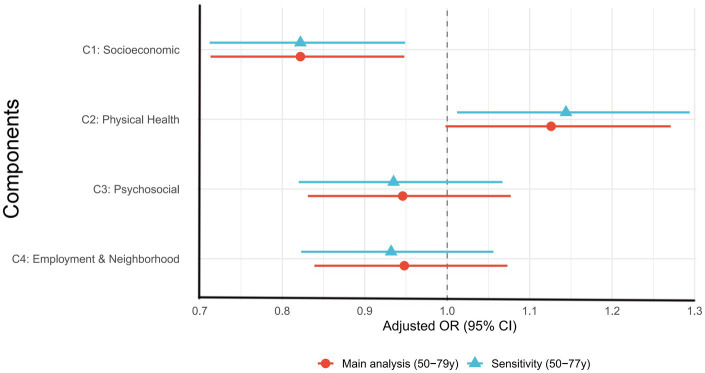
Sensitivity analysis: Excluding ages 78–79. A single logistic regression model including all four components, and all covariates simultaneously.

After excluding 197 participants aged 78–79 years (remaining n = 6,037), the core findings were consistent with the primary analysis: Socioeconomic Resources remained the sole significant predictor (OR = 0.822, 95% CI: 0.712–0.949, *p* = 0.007). A noteworthy change, however, was that Physical Health crossed the threshold of statistical significance (OR = 1.144, 95% CI: 1.012–1.294, *p* = 0.032), compared with its borderline status in the full sample (*p* = 0.053). This shift suggests that the inclusion of adults aged 78–79—who face a Medicare coverage gap for lung cancer screening, as CMS currently covers LDCT only for ages 50–77—may introduce a countervailing dynamic: individuals in this age range likely have high physical morbidity yet lack coverage, thereby potentially attenuating the positive association between poor health and screening uptake. Psychosocial Health (*p* = 0.319) and Employment & Neighborhood (*p* = 0.267) remained non-significant (see Figure 4).

Additionally, to address missingness in covariates and potential selection bias, we compared the complete case multivariable regression (*n* = 5,171) with the imputed model (*n* = 6,234) (Supplementary Table 2). The core findings remained highly robust. Socioeconomic Resources (C1) remained the primary and only independent barrier to screening in both the complete case analysis (OR = 0.822, 95% CI: 0.713–0.948, *p* = 0.007) and the imputed model (OR = 0.817, 95% CI: 0.719–0.929, *p* = 0.002). The estimates for Physical Health, Psychosocial Health, and Employment & Neighborhood remained consistent in direction and significance across both models.

## 4 Discussion

This study utilized a nationally representative sample from the 2024 BRFSS with complex survey weighting to examine the association of social determinants and health status with UTD-LCS uptake in high-risk adults across the United States. By integrating PCA with clustering and SHAP analyses, we explored the heterogeneous drivers. Our results reveal that the weighted UTD-LCS uptake was 20.0%, consistent with recent national estimates of 16%–18%, and that socioeconomic deprivation is the dominant independent barrier to screening. Crucially, our robust weighted analysis clarified that socioeconomic resource deprivation is the dominant independent barrier to screening, while physical morbidity acts as a primary facilitator, a relationship heavily modulated by Medicare coverage policies.

Our finding that Socioeconomic Resources (encompassing food insecurity, utility and bill payment difficulties, and lack of transportation) emerged as the dominant independent social barrier corroborates and significantly refines the recent literature on screening disparities. Recent analyses of national datasets, such as the 2025 studies by Do et al. (24) and Elkefi et al. (15), have highlighted that the accumulation of individual adverse SDOH—including housing insecurity and financial strain—correlates with severe underutilization of LCS. Similarly, recent comprehensive reviews emphasize that systemic disparities remain pervasive across the lung cancer care continuum (11, 14). However, while prior studies predominantly assessed these SDOH metrics as isolated variables or simple additive scores, our findings explicitly advance this paradigm. By distilling 14 complex variables into latent dimensions using PCA and evaluating them via SHAP, we demonstrate that structural material deprivation fundamentally eclipses psychosocial vulnerabilities (e.g., loneliness, lack of emotional support) in predicting screening behaviors. Unlike individual demographic factors, this latent dimension reflects a composite state of material deprivation. For highly vulnerable populations, preventive care often falls to the bottom of the priority list when competing against immediate survival needs, such as securing food or maintaining housing (25, 26). Furthermore, our results suggest that economic barriers operate holistically; interventions addressing only a single aspect (e.g., providing free transportation) may fail if the individual is concurrently overwhelmed by food insecurity or the fear of downstream financial toxicity associated with false-positive findings or subsequent lung cancer treatments (27, 28).

An important finding of this study relates to the complex role of Physical Health in driving screening behavior. Initially showing a borderline positive association (*p* = 0.053), Physical Health became definitively significant only after excluding participants aged 78–79. This finding highlights a discrepancy between clinical need and current coverage policies. Generally, individuals with greater physical morbidity interact more frequently with the healthcare system, creating more “teachable moments” for providers to identify eligibility and recommend LCS (15). However, the Centers for Medicare & Medicaid Services currently caps LCS coverage at age 77 (29). Thus, the 78–79 age cohort—who typically possess the highest chronic disease burden and theoretically the highest drive for screening—are abruptly subjected to high out-of-pocket costs, which may potentially offset the positive push of their physical morbidity (30). While based on indirect cross-sectional inference, these suggestive findings align with existing arguments advocating for the alignment of CMS screening coverage with broader USPSTF age guidelines (up to age 80). However, further longitudinal studies are needed to confirm whether the most clinically vulnerable are directly deterred by abrupt out-of-pocket costs (31).

Furthermore, the clustering and SHAP analyses revealed underlying heterogeneity in screening barriers despite similar overall rates. The PAM clustering algorithm divided the population into a “General” cluster and an “Employment/Neighborhood Vulnerable” cluster. Surprisingly, the overall screening rates between these two starkly different groups were nearly identical (20.2% vs. 18.7%). Conventional analyses might erroneously conclude that employment and neighborhood environments do not affect screening. However, our SHAP analysis demonstrated otherwise. In the Vulnerable cluster, the predictive contributions of both Physical Health (the facilitator) and Socioeconomic Resources (the barrier) were magnified two- to three-fold compared to the General population. This highlights conflicting factors among disadvantaged individuals: while physical morbidity drives healthcare utilization, severe socioeconomic deprivation acts as a counteracting barrier, resulting in stagnant overall screening rates (31, 32). The net result is a stagnant screening rate that masks intense underlying struggle.

The public health implications of these insights are substantial. They imply that universal, broad-stroke awareness campaigns will yield diminishing returns, especially for vulnerable populations whose screening decisions are caught in a structural gridlock. To improve LCS equity, precision public health strategies are required. For the general population, capitalizing on clinical encounters to recommend screening may suffice. For socially vulnerable groups, however, clinical recommendations must be tightly coupled with community-based interventions—such as patient navigation, mobile screening units, and direct assistance with insurance and basic social needs—to address competing socioeconomic barriers.

This study has several limitations. First, BRFSS data are self-reported and subject to recall and social desirability biases. Specifically, the survey instrument asks about ‘CT or CAT scans’ and cannot reliably distinguish whether respondents received a true guideline-concordant LDCT or a standard diagnostic chest CT. Second, the cross-sectional design precludes causal inference regarding the relationship between the identified latent dimensions and screening behavior. Third, individuals aged 80 and older were excluded due to data aggregation constraints, potentially omitting unique determinants present in the oldest-old population. Fourth, the exclusion of 11 states that did not administer the SDOH module may limit the generalizability of our findings to the entire United States, particularly if these non-participating states differ in socioeconomic composition. Individuals who declined to answer SDOH questions may be more socially vulnerable, potentially resulting in the exclusion of the very population this study aims to characterize. Fifth, the reclassification of the alcohol variable resulted in the exclusion of 88 participants with missing data, though this represents a small proportion (1.4%) of the sample. Sixth, the substantial sample reduction from the initial cohort due to missing data and module availability introduced systematic differences between the included and excluded populations, as shown in Supplementary Table 1. Specifically, our final sample underrepresented younger, uninsured, and unmarried individuals, which may introduce selection bias. However, assuming a Missing at Random (MAR) mechanism, our sensitivity analysis using multiple imputation yielded highly consistent results with the primary complete case analysis, suggesting that these missingness structures did not meaningfully bias our core finding regarding socioeconomic barriers. Furthermore, while the application of survey weights is essential for producing unbiased population estimates, it may reduce statistical power, as reflected in the borderline significance of Physical Health (*p* = 0.053). Finally, while SHAP values offer strong model interpretability, they explain algorithmic predictions rather than establishing biological or sociological causality. Despite these limitations, this study provides a novel, data-driven framework for understanding the nuanced and heterogeneous drivers of lung cancer screening, offering a roadmap for more targeted and equitable public health interventions.

## 5 Conclusion

In conclusion, UTD-LCS rates remain low and are hindered primarily by composite socioeconomic resource deprivation. While poor physical health generally prompts individuals to seek screening, this pathway appears to be potentially complicated by the Medicare coverage gap for adults aged 78–79. Furthermore, highly socially vulnerable individuals experience amplified, conflicting pressures regarding screening decisions that cannot be resolved through clinical advice alone. Advancing screening equity may benefit from exploring the alignment of CMS coverage policies with broader age guidelines and integrating robust socioeconomic support systems directly into the lung cancer screening pathway.

## Data Availability

The raw data supporting the conclusions of this article will be made available by the authors, without undue reservation.

## Supplementary material

SUPPLEMENTARY FIGURE S1 Scree plot.
