# Supplementary figures and images for: Who is more likely to receive up-to-date lung cancer screening? Identifying key barriers using principal component analysis and SHAP modeling: a weighted cross-sectional analysis of the 2024 BRFSS
Source: Front Public Health. 2026 Jun 18;14:1803742. doi: 10.3389/fpubh.2026.1803742 (PMC13323023; doi:10.3389/fpubh.2026.1803742)

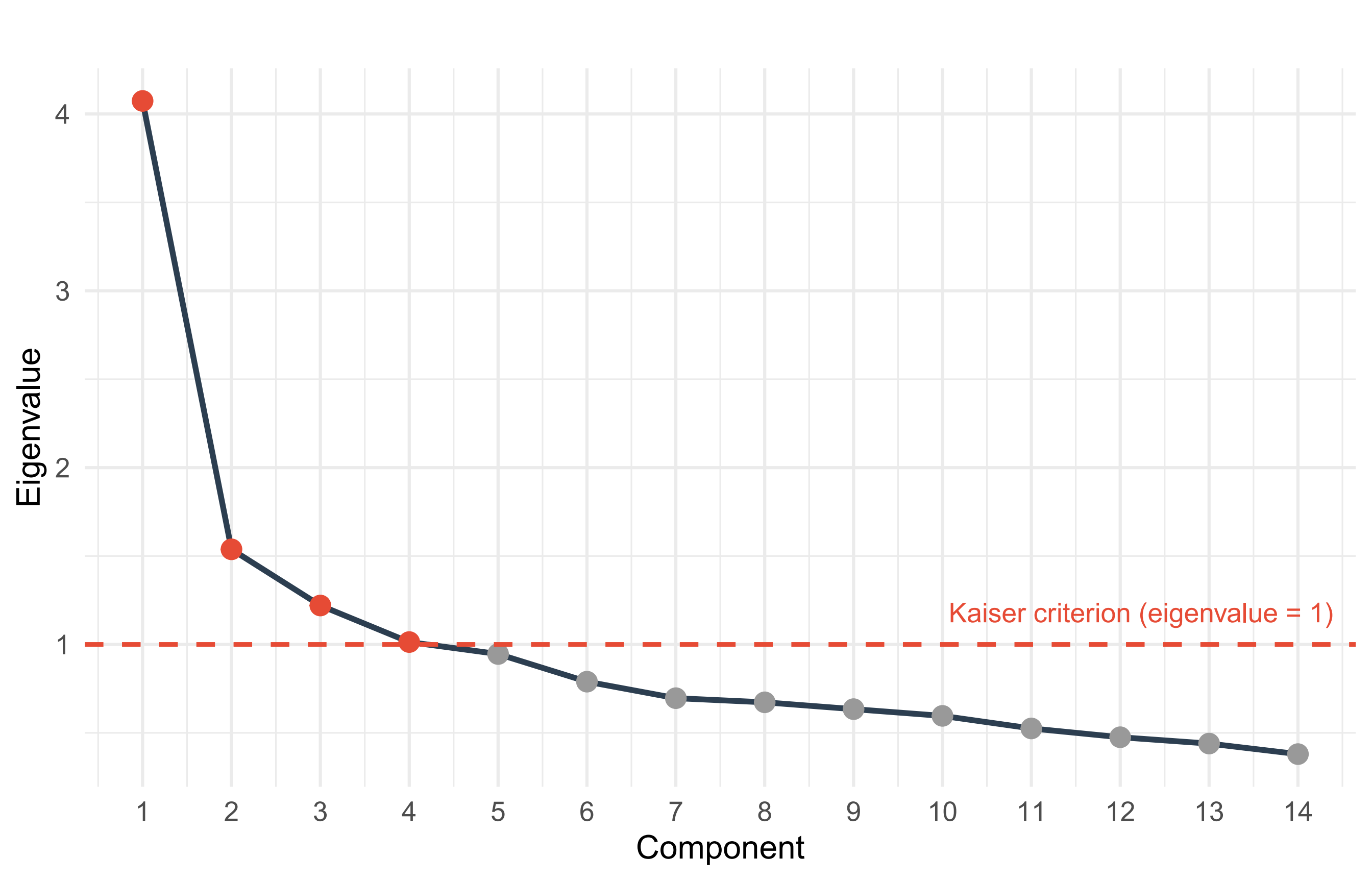

Supplement: SUPPLEMENTARY FIGURE S1 — Scree plot. [file Image_1.TIF]
