# Supplementary material for: Who is more likely to receive up-to-date lung cancer screening? Identifying key barriers using principal component analysis and SHAP modeling: a weighted cross-sectional analysis of the 2024 BRFSS
Source: Front Public Health. 2026 Jun 18;14:1803742. doi: 10.3389/fpubh.2026.1803742 (PMC13323023; doi:10.3389/fpubh.2026.1803742)
Supplement: Supplementary file 3 [file Table_1.docx]

**Supplementary Table 1.** Comparison of baseline characteristics between included and excluded populations (weighted)

| Variable | Total (N=37,651) | Included (n=6,234) | Excluded (n=31,417) | *P* | SMD |
| --- | --- | --- | --- | --- | --- |
| Age, year | 58.6 ± 12.0 | 63.5 ± 7.4 | 57.7 ± 12.4 | <0.001 | 0.566 |
| Sex, n (%) |  |  |  | 0.497 | -0.021 |
| Male | 19,878 (56.7) | 3,251 (55.8) | 16,627 (56.9) |  |  |
| Female | 17,773 (43.3) | 2,983 (44.2) | 14,790 (43.1) |  |  |
| Race, n (%) |  |  |  | 0.029 | 0.127 |
| White only, Non-Hispanic | 31,497 (76.4) | 5,287 (75.9) | 26,210 (76.5) |  |  |
| Black only, Non-Hispanic | 1,598 (7.2) | 373 (8.5) | 1,225 (7.0) |  |  |
| Other race only, Non-Hispanic | 1,496 (5.4) | 184 (7.0) | 1,312 (5.2) |  |  |
| Multiracial, Non-Hispanic | 1,022 (3.2) | 111 (2.5) | 911 (3.3) |  |  |
| Hispanic | 1,464 (7.7) | 202 (6.1) | 1,262 (8.0) |  |  |
| Education level, n (%) |  |  |  | 0.042 | 0.096 |
| Less than high school | 3,888 (16.8) | 551 (15.1) | 3,337 (17.1) |  |  |
| Graduated high school | 13,813 (34.6) | 2,207 (32.8) | 11,606 (34.9) |  |  |
| Attended college or technical school | 12,544 (34.9) | 2,118 (36.0) | 10,426 (34.7) |  |  |
| Graduated from college or technical school | 7,331 (13.7) | 1,350 (16.1) | 5,981 (13.3) |  |  |
| Annual Income, n (%) |  |  |  | 0.208 | 0.043 |
| < $50,000 | 18,870 (52.6) | 3,206 (54.4) | 15,664 (52.3) |  |  |
| ≥ $50,000 | 14,006 (47.4) | 2,316 (45.6) | 11,690 (47.7) |  |  |
| Health insurance, n (%) |  |  |  | <0.001 | 0.208 |
| No | 2,111 (7.6) | 166 (3.5) | 1,945 (8.3) |  |  |
| Yes | 34,378 (92.4) | 5,921 (96.5) | 28,457 (91.7) |  |  |
| Leisure time exercise, n (%) |  |  |  | 0.381 | -0.028 |
| No | 14,481 (37.6) | 2,425 (38.8) | 12,056 (37.4) |  |  |
| Yes | 23,033 (62.4) | 3,796 (61.2) | 19,237 (62.6) |  |  |
| Urban or Rural status, n (%) |  |  |  | 0.649 | 0.009 |
| Urban | 29,892 (89.9) | 5,125 (90.1) | 24,767 (89.8) |  |  |
| Rural | 6,925 (10.1) | 1,037 (9.9) | 5,888 (10.2) |  |  |
| Smoking status, n (%) |  |  |  | 0.172 | 0.060 |
| person who smokes daily | 18,886 (49.1) | 2,971 (46.6) | 15,915 (49.5) |  |  |
| person who smokes some days | 2,932 (7.7) | 505 (7.8) | 2,427 (7.7) |  |  |
| person who formerly smoked | 15,833 (43.2) | 2,758 (45.6) | 13,075 (42.8) |  |  |
| Marital status, n (%) |  |  |  | <0.001 | 0.122 |
| Married | 15,541 (46.6) | 2,812 (51.8) | 12,729 (45.7) |  |  |
| Others | 21,928 (53.4) | 3,403 (48.2) | 18,525 (54.3) |  |  |
| Alcohol use, n (%) |  |  |  | <0.001 | 0.121 |
| Non-drinker | 3,132 (9.2) | 416 (7.9) | 2,716 (9.4) |  |  |
| Moderate | 2,871 (9.2) | 397 (6.8) | 2,474 (9.6) |  |  |
| Binge/Heavy drinking | 30,295 (81.6) | 5,312 (85.3) | 24,983 (81.0) |  |  |

SMD: Standardized difference between included and excluded groups (based on weighted estimates, Mahalanobis-type for multiple categories); |SMD| ≥ 0.1 indicates imbalance between groups.

**Supplementary Table 2.** Robustness of primary results: Complete case analysis vs. multiple imputation for the four latent components (C1–C4)

| Variable | Complete case analysis (n=5,171) | | Multiple imputation (n=6,234) | |
| --- | --- | --- | --- | --- |
|  | OR (95% CI) | *P*-value | OR (95% CI) | *P*-value |
| Principal component scores |  |  |  |  |
| Socioeconomic resources | 0.822 (0.713, 0.948) | 0.007 | 0.817 (0.719, 0.929) | 0.002 |
| Physical health | 1.126 (0.998, 1.271) | 0.053 | 1.114 (0.999, 1.243) | 0.053 |
| Psychosocial health | 0.946 (0.831, 1.077) | 0.403 | 0.960 (0.856, 1.077) | 0.491 |
| Employment & Neighborhood | 0.948 (0.839, 1.073) | 0.399 | 0.968 (0.839, 1.078) | 0.553 |

Abbreviations: OR, odds ratio; CI, confidence interval
